# TNF-ɑ Induces Methylglyoxal Accumulation in Lumbar Herniated Disc of Patients With Radicular Pain

**DOI:** 10.3389/fnbeh.2021.760547

**Published:** 2021-11-23

**Authors:** Xinsheng Zhang, Xiaogang Wang, Liang Gao, Bin Yang, Yahan Wang, Kerun Niu, Jiahui Lai, Shun Wan, Jianping Luo

**Affiliations:** ^1^Spinal Surgery Department, Henan Provincial People’s Hospital, Zhengzhou University, Zhengzhou, China; ^2^Hua Tuo Institute of Medical Innovation, Wuhan, China; ^3^Sino Euro Orthopaedics Network, Berlin, Germany; ^4^Department of Orthopaedic, Henan Provincial People’s Hospital, Zhengzhou University, Zhengzhou, China; ^5^Medical School, Henan University, Kaifeng, China; ^6^Medical School, Zhengzhou University, Zhengzhou, China

**Keywords:** lumbar disc herniation, methylglyoxal, TNF-ɑ, pain, GLO1

## Abstract

Lumbar disc herniation (LDH) with radicular pain is a common and complicated musculoskeletal disorder. Our previous study showed that LDH-induced methylglyoxal (MG) accumulation contributed to radicular pain. The underlying mechanisms through which MG accumulates are poorly understood. In the present study, we found that both MG and tumor necrosis factor-alpha (TNF-ɑ) levels in the herniated disc of patients with radicular pain were significantly increased, and the activity of Glyoxalase 1 (GLO1), the rate-limiting enzyme that metabolizes MG, was decreased. In rats, the LDH model was mimicked by implantation of autologous nucleus pulposus (NP) to the left lumbar five spinal nerve root. The mechanical allodynia was observed in LDH rats. Besides, MG and TNF-ɑ levels were increased, and GLO1 activity was significantly decreased in the implanted NP. In cultured rat NP cells, stimulation with the inflammatory mediator TNF-ɑ reduced GLO1 activity and expression. These results suggested that TNF-ɑ-induced GLO1 activity decrease contributed to MG accumulation in the herniated disc of patients with radicular pain.

## Introduction

Lumbar radicular pain after intervertebral disc herniation is one of the most prevalent causes of physical disability. It is caused not solely by mechanical compression of the nerve root but also by the release of many inflammatory molecules (Takahashi et al., 1996; Ahn et al., 2002; Scuderi et al., 2006; Pedersen et al., 2015). Clinical data indicate that 20–76% of nerve root compression due to a disc herniation is painless, and some cases with slight disc herniation suffer severe pain (Takada et al., 2001; Djuric et al., 2020). Therefore, a chemical factor may play an important role in radicular leg pain, following lumbar disc herniation (Andrade et al., 2013; Hasvik et al., 2019; Wang et al., 2020). Experimental studies indicate that inflammatory molecules are released from leaked nucleus pulposus (NP) and attracted immune cells, which include tumor necrosis factor-alpha (TNF-ɑ), interleukin-6 (IL-6), interleukin-8 (IL-8), metalloproteinases, cyclooxygenase-2, nitric oxide, and so on (Andrade et al., 2011; Pedersen et al., 2015; Djuric et al., 2020).

The intervertebral disc is the largest avascular and immune-privileged tissue in our body. Oxygen and glucose diffused into the disc can be exhausted when nucleus pulposus material degenerates or herniates into the epidural space (Guehring et al., 2009). Therefore, glycolysis has been enhanced as the main source of energy for disc cells. Methylglyoxal (MG), as the reactive glycolytic by-product, has serious toxicological effects when it is excessively accumulated. Increased MG was found in cerebrospinal fluid of patients with Alzheimer’s disease, and this was associated with poorer cognitive function and lower brain volume (Angeloni et al., 2014). The levels of plasma MG in patients who experienced diabetic pain were significantly higher than those in patients with diabetes without pain (Bierhaus et al., 2012). Previous data from our group suggest patients who suffered from a lumbar disc herniation (LDH)-induced pain had elevated plasma methylglyoxal (MG) levels and increased MG in dorsal root ganglions (DRG)-induced radicular pain in a rat model of lumbar disc herniation (Liu et al., 2017). It is well-known that the glyoxalase system is the main enzyme that metabolizes MG, especially GLO1, as the rate-limiting step of this series of reactions uses L-glutathione (GSH) as a cofactor (Gaffney et al., 2020). Previous studies have shown that GLO1 levels and activity can be altered in disease states, including diabetes, cardiomyopathy, and endothelial dysfunction (Jack et al., 2012; Skapare et al., 2013; Hanssen et al., 2014; Yumnam et al., 2020). Therefore, both GLO1 and GSH are key factors in maintaining MG at low tolerable levels, preventing protein and cell dysfunction. However, we still do not know where MG is released from and how it increases in patients with LDH with radicular pain. Herein, we hypothesize that, when NP material herniates into the epidural space, inflammation factors including TNF-ɑ are released, which decreases GLO1 activity and increases the MG level in a herniated disc.

## Materials and Methods

### Patients and Volunteers

This study was approved by the Ethics Committee at Henan Provincial People’s Hospital, Zhengzhou University. Thirty patients were prospectively enrolled at the Spinal Surgery Department and included 20 patients with LDH, suffering from radicular leg pain for less than 3 months and 10 patients with scoliosis or lumbar burst fracture as the control without leg painful symptomatology or degenerative disc disease. The LDH patient group had a mean age of 43 years and consisted of 11 men and 9 women. The control patient group had a mean age of 36 years and consisted of seven men and three women. Patients with LDH had assessed the intensity of leg pain on a 0–10 (0, no pain; 10, worst pain) visual analog scale (VAS) 1 day before discectomy. Patients with LDH were divided into two groups according to their preoperative pain scores (VAS ≤ 3 as mild pain group; VAS ≥ 4 as severe pain group). Intraoperative-collected herniated disc (HD) tissues, obtained during discectomy in patients with LDH and during orthopedic surgery in control patients, were collected. Immediately upon collection, tissues were divided into two parts. One part was flash frozen in liquid nitrogen and stored at −80°C for further use, and the other part was fixed in 4% formaldehyde solution for histopathological assessment.

### Animals and Surgery

Male Sprague Dawley rats (200–220 g) were obtained from the Institute of Experimental Animals of Zhengzhou University. All rats were housed in a temperature- and humidity-controlled environment on a 12/12-h light/dark cycle and provided with food and water *ad libitum*. All experimental procedures were approved by the Institutional Animal Care Committee of Zhengzhou University and were carried out in accordance with the guidelines of the National Institutes of Health Guide for the care and use of laboratory animals. Efforts were made to minimize animal suffering and to reduce the number of animals used.

Surgery for the lumbar disc herniation model was performed as previously described by Anzai et al. (2002) and Liu et al. (2017). In brief, rats were anesthetized intraperitoneally with sodium pentobarbital (50 mg/kg), and laminectomies were performed in which the left L5 nerve roots and corresponding dorsal root ganglion (DRG) were exposed. Autologous NP harvested from the coccygeal intervertebral disc was applied to the left L5 nerve roots just proximal to the corresponding DRG. The surgical procedure in the sham group was identical to the LDH group except for the application of NP to the left L5 nerve roots. TNF-ɑ inhibitor (500 μg Etanercept) was injected into the implanted NP after the application of NP to the left L5 nerve roots. The dose of Etanercept was determined based on the results from previous experiments (Horii et al., 2011; Inage et al., 2016). Special care was taken to prevent infection and minimize the influence of inflammation.

### Behavioral Test

The 50% withdrawal threshold was assessed using von Frey hairs as described previously (Chaplan et al., 1994). Briefly, each rat was loosely restrained beneath a plastic box on a metal mesh for at least 15 min one time daily for 3 separate days, and mechanical allodynia in the LDH and sham groups were examined 1 day before surgery. Following 7 days of recovery, the test was performed weekly until 4 weeks postoperatively. Mechanical allodynia was assessed by the hind paw withdrawal threshold in response to probing with a series of von Frey filaments (bending force from 0.55 to 20.30 g) for 6 s or until the rat withdrew. A nociceptive response was defined as a brisk paw withdrawal or flinching of the paw, following von Frey filament application. Each test was repeated two to three times at approximately 2 min intervals, and the average value of von Frey filament force was determined as the force to evoke a withdrawal response. The experimenter who conducted the behavioral test was blinded to all treatments.

### Western Blot

The herniated disc tissue was collected and immediately stored at −80°C until use. The tissue was homogenized on ice. Protein samples were separated by gel electrophoresis (SDS-PAGE) and transferred onto a PVDF membrane. The blots were incubated with a primary antibody against GLO1 (1:200, ABCAM, United States) and β-actin (1:2,000, Cell Signaling Technology, United States) overnight at 4°C according to the instructions of the manufacturer. The blots were then incubated with a secondary antibody. ECL (Pierce, United States) was used to detect the immune complex. After exposure for 2 min, the bands were achieved under Chemiluminescence and Fluorescence Imaging System (G:BOX XT4, Syngene, United Kingdom). The bands were quantified with a computer-assisted imaging analysis system (NIH Image J).

### Culture of Nucleus Pulposus Cells

Nucleus pulposus cells were collected from the lumbar disc of 10 male Sprague-Dawley rats (3 months old). All experimental procedures described below were reviewed and approved by the Ethics Committee at Henan Provincial Peoples’ Hospital, Zhengzhou University, China. In brief, the rats were killed by an intraperitoneal overdose injection of 10% chloral hydrate, and the NP tissue was collected from the coccygeal intervertebral disc under aseptic conditions. After cutting the tissues into 1 × 1 mm^3^ sections, 0.2% type 2 collagenase (Sigma-Aldrich, St. Louis, MO, United States) was added and digested for 4 h. After washing with phosphate-buffered saline (PBS) and centrifuging for 5 min at 1,500 *g*, the isolated cells were cultured in Dulbecco’s modified Eagle’s medium (DMEM) with 10% fetal bovine serum (FBS) and antibiotics (100 U/ml penicillin and 100 U/ml streptomycins) at 37°C in a 5% CO_2_ incubator. The total number of cells was less than 1,000. NP cells from the second passage were treated with different doses of TNF-ɑ.

### Glyoxalase 1 (GLO1) Activity Assays

Nucleus pulposus material was dissected on dry ice and stored at −80°C until use. The samples were homogenized, and the supernatant was collected for further assays. The total protein concentration was determined using the BCA Protein Assay Kit (Pierce, United States). GLO1 activity was measured as described by Hovatta et al. (2005) and the Glyoxalase 1 Assay Kit (Sigma-Aldrich, United States) according to the instructions of the manufacturer. The GLO1 activity rate was calculated by the absorbance at 240 nm.

### Reduced/Oxidized Glutathione (GSH/GSSG) Ratio Detection Assays

The GSH/GSSG ratio was measured by using the described method with a minor modification (Baig et al., 2020). Briefly, NP material was dissected on dry ice and stored at −80°C until use. The samples were homogenized in 1.5 ml of a cold homogenization buffer for 1 min, and the supernatant was collected for further assays. GSH and GSSG were quantified on a fluorescent microplate reader at an excitation/emission wavelength set to 490/520 nm. Absolute amounts of GSH and GSSG were determined using GSH and GSSG standard curves.

### Methylglyoxal (MG) Determination by HPLC

The concentration of methylglyoxal was determined by HPLC using a simple derivatization procedure (Liu et al., 2017). Briefly, NP material was homogenized on dry ice, and the supernatant sample was supplemented with internal standard 5-methylquinoxaline (5-MQ) and the o-phenylenediamine (o-PD) at room temperature for 4 h. Perchlorate (PCA) was added to the derived sample and incubated on ice for 10 min. Methylglyoxal (2-MQ) and the quinoxaline internal standard (5-MQ) were measured using the conditions below. The analysis conditions were applied as follows: detector wavelength, 315 nm; mobile phase flow rate, 1. ml/min; typical sample size, 15 μl; and column temperature, 20°C. Duplicate injections of each sample were made. Samples were calibrated by comparison with a 2-MQ standard. The average retention times of 2-MQ and 5-MQ were 3.76 and 7.55 min, respectively.

### Statistical Analysis

All results are statistically confirmed SPSS 13.0 (SPSS, United States) and expressed as mean ± SEM. Statistical differences between the two groups were analyzed by one-way ANOVA. One-way or two-way ANOVA with repeated measures followed by Tukey, Dunnett, or Bonferroni *post hoc* test was carried out to compare differences between more than two groups. The criterion for statistical significance was *p* < 0.05. Complete statistical analysis is detailed in figure legends.

## Results

### Increased Methylglyoxal Levels in Herniated Disc Contribute to Radicular Pain Induced by Lumbar Disc Herniation

**FIGURE 1:**
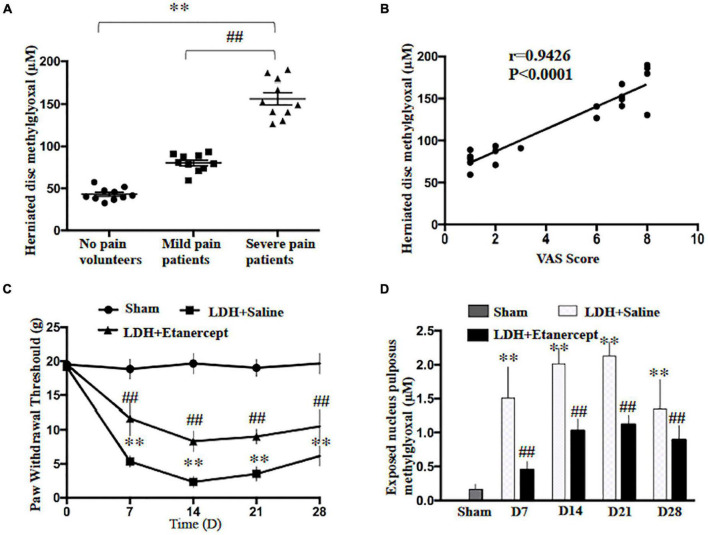
Lumbar-herniated disc (LDH)–induced radicular pain and methylglyoxal (MG) accumulation in herniated disc. **(A)** MG levels were evaluated in herniated disc of the patients or volunteers (One-way ANOVA: *F* = 143.679, *p* < 0.001; *post hoc* Dunnett: ^∗∗^*p* < 0.001, volunteers with no pain vs. patients with severe pain. ##*p* < 0.001 patients with mild pain vs. patients with severe pain, *n* = 10). **(B)** The analysis of linear correlation between visual analog scale (VAS) and the herniated disc MG level in patients. **(C)** The paw withdrawal threshold of rats was significantly decreased following NP implantation (Two-way ANOVA: *F* = 3.893, *p* = 0.004. *Post hoc* Tukey; sham vs. LDH + Saline at 7, 14, 21, 28 days: ^∗∗^*p* < 0.001. Lumbar-herniated disc (LDH) + Etanercept vs. LDH + Saline at 7, 14, and 21 days: ##*p* < 0.001, at 28 days: ##*p* = 0.002. *n* = 6). **(D)** The MG level of exposed NP was examined at different time points following NP implantation (One-way ANOVA: *F* = 37.061, *p* < 0.001. *Post hoc* Dunnett: sham vs. LDH + Saline at 14 and 21 days: ^∗∗^*p* < 0.001, at 7 days: ^∗∗^*p* = 0.005, at 28 days: ^∗∗^*p* = 0.007. One-way ANOVA, LDH + Etanercept vs. LDH + Saline: *F* = 29.944, ##*p* < 0.001 at 7 days; *F* = 71.625, ##*p* < 0.001 at 14 days; *F* = 54.421, ##*p* < 0.001 at 21 days; *F* = 5.464, ##*p* = 0.042 at 28 days. *n* = 6).

In the present study, we first found that the methylglyoxal levels of the herniated disc were significantly increased in the patients who suffered from the radicular leg pain accompanied by LDH compared with the patients with no-leg pain (Figure 1A). The analysis of Pearson correlation showed a strong positive linear correlation between VAS scores and a herniated disc MG level in patients with LDH (Figure 1B). To further investigate the causal relationship between MG- and LDH-induced radicular pain, the mechanical withdrawal threshold and the MG level in exposed NP tissues were examined in a rat NP implantation-induced LDH model. These animals exhibited significant mechanical allodynia on Days 7, 14, 21, and 28 after NP implantation (Figure 1C). Meanwhile, the MG level in exposed NP tissues was also significantly increased compared with that in the native NP tissue (Figure 1D). Note that the time course of increased MG was consistent with that of mechanical allodynia.

### GLO1 Activity Decrease Contributes to Excessive Accumulation of Methylglyoxal in the Herniated Disc

**FIGURE 2:**
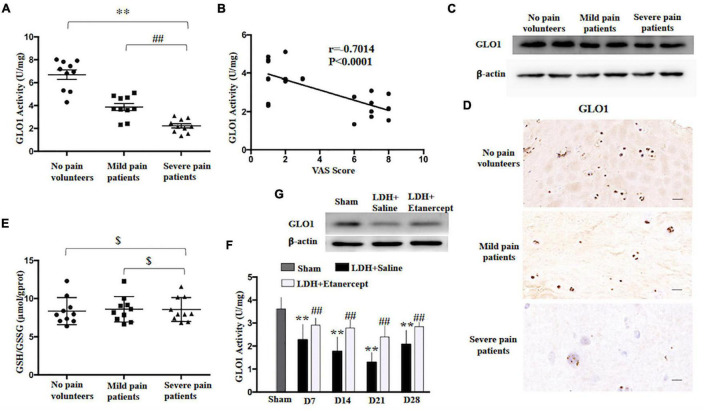
Glyoxalase 1 (GLO1) activity decrease contributes to excessively accumulation of MG in herniated disc. **(A)** GLO1 activity was evaluated in herniated disc of the patients or volunteers (one-way ANOVA: *F* = 51.276, *p* < 0.001; *post hoc* Bonferroni: ^∗∗^*p* < 0.001, volunteers with no pain vs. patients with severe pain. ##*p* = 0.003 patients with mild pain vs. patients with severe pain, *n* = 10). **(B)** The analysis of linear correlation between VAS and herniated disc GLO1 activity in patients. **(C,D)** Representative immunoblotting and immunohistochemistry showed the downregulation of GLO1 in herniated disc of patients. **(E)** GSH/GSSG was evaluated in herniated disc of the patients or volunteers (One-way ANOVA: *F* = 0.062, $ *p* = 0.94 > 0.05. *n* = 10). **(F)** The GLO1 activity of exposed NP was evaluated at different time points, following NP implantation (one-way ANOVA: *F* = 13.986, *p* < 0.001. *Post hoc* Bonferroni: sham vs. LDH + Saline at 14 days and 21 days: ^∗∗^*p* < 0.001, at 7 days: ^∗∗^*p* = 0.003, at 28 days: ^∗∗^*p* = 0.001. One-way ANOVA, LDH + Etanercept vs. LDH + Saline: *F* = 8.116, ##*p* = 0.017 at 7 days; *F* = 12.039, ##*p* = 0.006 at 14 days; *F* = 17.944, ##*p* = 0.002 at 21 days; *F* = 8.866, ##*p* = 0.014 at 28 days. *n* = 6). **(G)** Representative immunoblotting showed the downregulation of GLO1 in exposed NP of rats on Day 14 after NP implantation.

It is well known that the glyoxalase system in the cytoplasm is the main enzyme that metabolizes methylglyoxal and Glo1 as the rate-limiting enzyme and uses L-glutathione (GSH) as a cofactor (Gaffney et al., 2020). In the present study, we found that GLO1 activity of HD decreased in the patients who suffered from the LDH-induced radicular pain compared to the patients with no-leg pain (Figure 2A). The analysis of Pearson correlation showed a strong negative linear correlation between VAS scores and herniated disc GLO1 activity in patients with LDH (Figure 2B). In comparison with the patients with no-leg pain, immunoblotting and immunohistochemistry showed GLO1 expression decreased significantly in patients with radicular pain (Figures 2C,D). However, for GSH, there was no significant difference between patients with LDH-induced radicular pain and patients with no-leg pain (Figure 2E). Moreover, both GLO1 activity and expression decreased in the rats with NP implantation compared with that in the native NP tissue (Figures 2F,G).

### TNF-ɑ Induced Methylglyoxal Accumulation Through Reducing GLO1 Activity in the Herniated Disc and Cultured Nucleus Pulposus Cell

**FIGURE 3:**
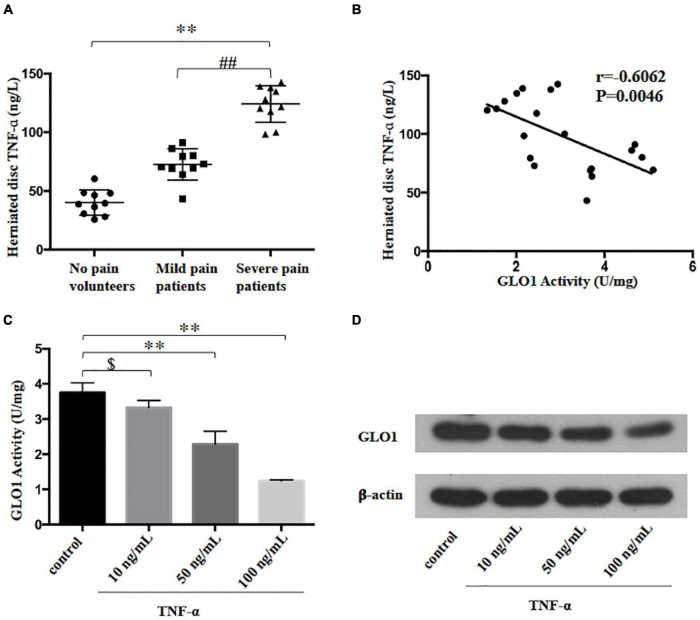
Tumor necrosis factor-alpha (TNF-ɑ)-reduced GLO1 activity and expression in herniated disc and cultured nucleus pulposus cell. **(A)** TNF-ɑ levels were evaluated in herniated disc of the patients or volunteers (One-way ANOVA: *F* = 100.443, *p* < 0.001; *post hoc* Bonferroni: ^∗∗^*p* < 0.001, volunteer with no pain vs. patients with severe pain. ##*p* < 0.001, patients with mild pain vs. patients with severe pain, *n* = 10). **(B)** The analysis of linear correlation between TNF-ɑ levels and GLO1 activity in herniated disc of patients. **(C,D)** Both GLO1 activity and expression of NP cells were decreased by different doses of TNF-ɑ (One-way ANOVA: *F* = 58.312, *p* < 0.001; *post hoc* Bonferroni: control vs. 10 ng/ml TNF-ɑ: $ *p* = 0.446; control vs. 50 ng/ml TNF-ɑ: ^∗∗^*p* = 0.001; control vs. 100 ng/ml TNF-ɑ: ^∗∗^*p* < 0.001).

Research showed that stimulation with the inflammatory mediator TNF-ɑ reduced GLO1 activity in human U937 monocytes (Hanssen et al., 2014). In the present study, we found that TNF-ɑ level increased in the patients who suffered from the radicular leg pain, accompanied by LDH compared to the patients with no-leg pain (Figure 3A). Meanwhile, the analysis of Pearson correlation showed a strong negative linear correlation between TNF-ɑ and GLO1 activity in HD (Figure 3B). To further investigate the relation between TNF-ɑ and GLO1 activity, the primary NP cells were cultured. The GLO1 activity and expression of NP cells exposed to TNF-ɑ were decreased significantly compared with that in the control group (Figures 3C,D). Furthermore, etanercept treatment (500 μg), a known TNF-ɑ inhibitor, significantly inhibited the mechanical allodynia induced by NP implantation in the LDH rat model (Figure 1C). Meanwhile, etanercept treatment (500 μg) also significantly attenuated the decrease of GLO1 activity and expression and the increase of MG in the implanted NP of LDH rat (Figures 1D, 2F,G).

## Discussion

Previous studies have shown that MG, as a reactive byproduct of several metabolic pathways in cells, has been linked to painful neuropathies (Bierhaus et al., 2012; Ciobanu et al., 2016; Barragan-Iglesias et al., 2019). Our previous study also showed that MG accumulation contributed to radicular leg pain in patients with LDH and the NP implantation-induced LDH rat model (Liu et al., 2017). But, it is still unknown that where MG is released from and how it increases. In the present study, we found that the MG level in HD of patients who suffered radicular leg pain was significantly higher than that in the patients with no-leg pain. In addition, the MG level in herniated disc positively correlated with a leg VAS score in patients with LDH. Moreover, this phenomenon was verified in the NP implantation-induced LDH rat model. Hence, it is reasoned that MG may be released from the HD.

It is well-known that the glyoxalase system is the main enzyme that metabolizes MG to D-lactate, which is composed of two enzymes, glyoxalase 1 (GLO1) and GLO2. GLO1 is the rate-limiting step of this series of reactions, which uses L-glutathione (GSH) as a cofactor (Gaffney et al., 2020). GLO1 levels and activity can be altered in disease states, including diabetes, cardiomyopathy, and endothelial dysfunction (Jack et al., 2012; Skapare et al., 2013; Hanssen et al., 2014; Yumnam et al., 2020). Therefore, both GLO1 and GSH are key factors in maintaining MG at low tolerable levels, preventing protein and cell dysfunction. A recent study comparing the expression of GLO1 in various inbred mouse strains showed a negative correlation between GLO1 expression and mechanical hyperalgesia, implying that GLO1 might be linked to painful neuropathies (Jack et al., 2012). In the present study, we found that GLO1 activity and expression decreased significantly in the herniated disc of patients with radicular leg pain compared to that in patients with no-leg pain, and there was no difference between groups for GSH. We further found that the increased MG level in HD of patients and exposed NP of the rat model was concurrent with the decreases of GLO1 activity and expression. Therefore, GLO1 activity decrease may contribute to MG accumulation in a herniated disc.

When the immune-privileged nucleus pulposus migrates out of the normal intervertebral space, an inflammation reaction occurs (Shamji et al., 2010; Takada et al., 2012; Djuric et al., 2020). Various cytokines have been reported in disc biopsy samples from patients with LDH and experimental models (Takahashi et al., 1996; Shamji et al., 2010; Hiyama et al., 2021); among these, TNF-ɑ levels in herniated nucleus pulposus correlate with preoperative pain in patients with LDH (Genevay et al., 2008; Andrade et al., 2016). Rat models showed that the application of a TNF-ɑ inhibitor (etanercept) after disc puncture could decrease mechanical allodynia and downregulate the neuroinflammation factors (Horii et al., 2011). Therefore, we could speculate that an early vicious cycle created by TNF-ɑ-producing pain is perpetuated by different players. Recently, studies have shown that treatment with inflammatory cytokines, such as TNF decreased GLO1 activity in U937 monocytes, which suggested that inflammatory response may be involved in the onset and maintenance of MG excessive accumulation (Hanssen et al., 2014). So, it is possible to reason that TNF-ɑ secreted from herniated nucleus pulposus cells or immune cells reduced the GLO1 activity, which leads to the MG excessive accumulation. In our data, TNF-ɑ expression negatively correlated with GLO1 activity in herniated nucleus pulposus from patients with LDH and exposed NP of the rat model. In cultured nucleus pulposus cells, TNF-ɑ treatment decreased GLO1 activity and increased the accumulation of MG.

Taken together, our study supplies unique data, showing an association between TNF-ɑ and GLO1/MG in a herniated disc. When nucleus pulposus herniates, TNF-ɑ produced from herniated tissue or inflammatory cells may reduce GLO1 expression and activity in herniated nucleus pulposus, which increases the accumulation of MG, eventually inducing radicular pain. However, the TNF-ɑ/GLO1/MG pathway-involved mechanisms underlying radicular pain in the LDH need further investigation.

## Data Availability Statement

The raw data supporting the conclusions of this article will be made available by the authors, without undue reservation.

## Ethics Statement

The studies involving human participants were reviewed and approved by the Ethics Committee at Henan Provincial People’s Hospital, Zhengzhou University. The patients/participants provided their written informed consent to participate in this study. The animal study was reviewed and approved by the Institutional Animal Care Committee of Zhengzhou University.

## Author Contributions

XZ conceived the study, participated in its design, carried out the experiment of HPLC, western blot, nucleus pulposus cell culture, and drafted the manuscript. JLu conceived the study, participated in the design, and revised the manuscript. XW, BY, and YW carried out the collection of biopsies. LG and KN performed the statistical analysis and helped to revise the manuscript. JLa and SW carried out the behavioral tests, GLO1 activity assays, and GSH/GSSG assays. All authors contributed to the article and approved the submitted version.

## Conflict of Interest

The authors declare that the research was conducted in the absence of any commercial or financial relationships that could be construed as a potential conflict of interest.

## Publisher’s Note

All claims expressed in this article are solely those of the authors and do not necessarily represent those of their affiliated organizations, or those of the publisher, the editors and the reviewers. Any product that may be evaluated in this article, or claim that may be made by its manufacturer, is not guaranteed or endorsed by the publisher.
